# Nanoparticles with photoinduced precipitation for the extraction of pollutants from water and soil

**DOI:** 10.1038/ncomms8765

**Published:** 2015-07-21

**Authors:** Ferdinand Brandl, Nicolas Bertrand, Eliana Martins Lima, Robert Langer

**Affiliations:** 1 David H. Koch Institute for Integrative Cancer Research, Massachusetts Institute of Technology (MIT), 500 Main Street, Building 76-661, Cambridge, Massachusetts 02139, USA; 2 Laboratory of Pharmaceutical Technology, Federal University of Goiás, Goiânia, Goiás, Brazil; 3 Harvard-MIT Division of Health Sciences and Technology, and Department of Chemical Engineering, MIT, Cambridge, Massachusetts 02139, USA

## Abstract

Nanotechnology may offer fast and effective solutions for environmental clean-up. Herein, amphiphilic diblock copolymers are used to develop a platform of photosensitive core-shell nanoparticles. Irradiation with ultraviolet light removes the protective layer responsible for colloidal stability; as a result, the nanoparticles are rapidly and irreversibly converted to macroscopic aggregates. The associated phase separation allows measuring the partitioning of small molecules between the aqueous phase and nanoparticles; data suggests that interactions are enhanced by decreasing the particle size. Adsorption onto nanoparticles can be exploited to efficiently remove hydrophobic pollutants from water and contaminated soil. Preliminary *in vivo* experiments suggest that treatment with photocleavable nanoparticles can significantly reduce the teratogenicity of bisphenol A, triclosan and 17α-ethinyl estradiol without generating obviously toxic byproducts. Small-scale pilot experiments on wastewater, thermal printing paper and contaminated soil demonstrate the applicability of the approach.

## Abstract (web summary)

Nanoparticles can act as absorbent materials for environmental clean-up due to their high surface-to-volume ratio, but subsequent removal can be difficult. Here, the authors report nanoparticles that aggregate upon UV radiation, allowing them to absorb pollutants from water and subsequently be removed in the aggregated state.

## Graphical abstract

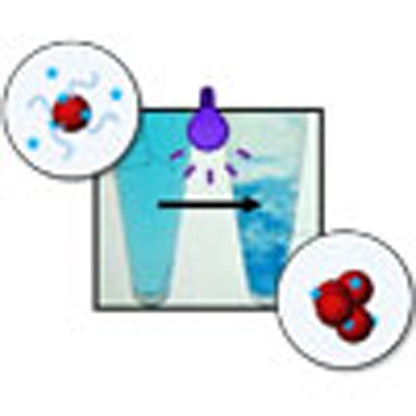

Exposure to certain chemicals, such as polychlorinated biphenyls, pesticides and certain endocrine disrupting compounds is associated with increased predisposition to cancer, diabetes, obesity, infertility and other endocrine disorders^1^,^2^,^3^. Although the precise impact of these chemicals on the prevalence of diseases is still unknown^4^, precautionary measures to minimize the release of chemicals into the environment are recommended. While waste avoidance and proper management of waste streams would be among the most sustainable practices, the high prevalence of contaminated waters and soils requires the development of new strategies for environmental remediation.

Nanotechnology may offer fast and effective solutions for environmental clean-up^5^. For example, nanostructured membranes with size-selective pores may provide efficient ways of separating solutes from water^6^. Besides filtration, which is generally energy-intensive, the removal of contaminants by sequestration (adsorptive remediation) or degradation to less toxic products (reactive remediation) may represent an effective alternative. Nanomaterials possess a very large surface-to-volume ratio that favours interaction with their environment. For example, nanomaterials have the potential to effectively adsorb molecules or catalyse chemical reactions at their interface^7^,^8^. A prominent example of a nanostructured material capable of removing contaminants by sequestration is known as self-assembled monolayers on mesoporous supports (SAMMS). SAMMS are formed by self-assembly of surfactants onto mesoporous ceramics; the extremely high inner surface area enables efficient extraction of heavy metals from aqueous and non-aqueous liquids^9^. For soil remediation, colloidal suspensions of amphiphilic nanomaterials are preferred to allow percolation through the sediment. In contrast to surfactant micelles, polymeric nanoparticles are stable at all concentrations and effective in desorbing hydrophobic contaminants from soil particles^10^,^11^. The most advanced remediation technology relying on the degradation of contaminants uses nanoparticles containing zero-valent iron or magnesium^12^. These nanoparticles are injected in soils or aquifers; they can remediate polychlorinated biphenyls and other chlorinated compounds by reduction at the water–metal interface. Two concerns possibly hindering the application of this technology are the formation of potentially toxic secondary products and the persistence of the nanomaterial in the environment^12^. Given the unknown health and ecological risks that this technology might have, finding effective ways of separating the nanomaterial from the treated sample is of particular importance. To enable separation, the use of paramagnetic Fe_3_O_4_@TiO_2_ core-shell nanoparticles has been proposed^13^,^14^. The TiO_2_ shell acts as photocatalyst for the degradation of organic pollutants in water; the Fe_3_O_4_ core allows for the magnetic separation from the dispersion. However, the long ultraviolet irradiation times (>60 min) required for the photooxidation of contaminants, the presence of inorganic non-degradable nanomaterials (TiO_2_ shell) and the need to introduce large magnets in the existing water purification infrastructures might complicate the application of this technology on a large scale.

Herein, we develop photo-responsive, biodegradable core-shell nanoparticles that combine several of the aforementioned design principles to bind and extract chemicals from contaminated waters and soils. The nanoparticles are prepared by self-assembly of amphiphilic diblock copolymers; the hydrophobic core of the nanoparticles acts as ‘trap' for hydrophobic molecules while the hydrophilic corona stabilizes the system. On irradiation with ultraviolet light, the nanoparticles shed their stabilizing layer, lose their colloidal stability and form macroscopic aggregates. These aggregates are enriched with the pollutant and can be easily separated (for example, by sedimentation and decantation, centrifugation or filtration). Irradiation with ultraviolet light is not required to sequester the pollutant; however, it provides a precipitation ‘switch' that allows exploiting the colloidal stability and large surface area of nanoparticles together with the safety and easy handling of bulkier materials.

## Results

### Preparation and characterization of nanoparticles

**Figure 1:**
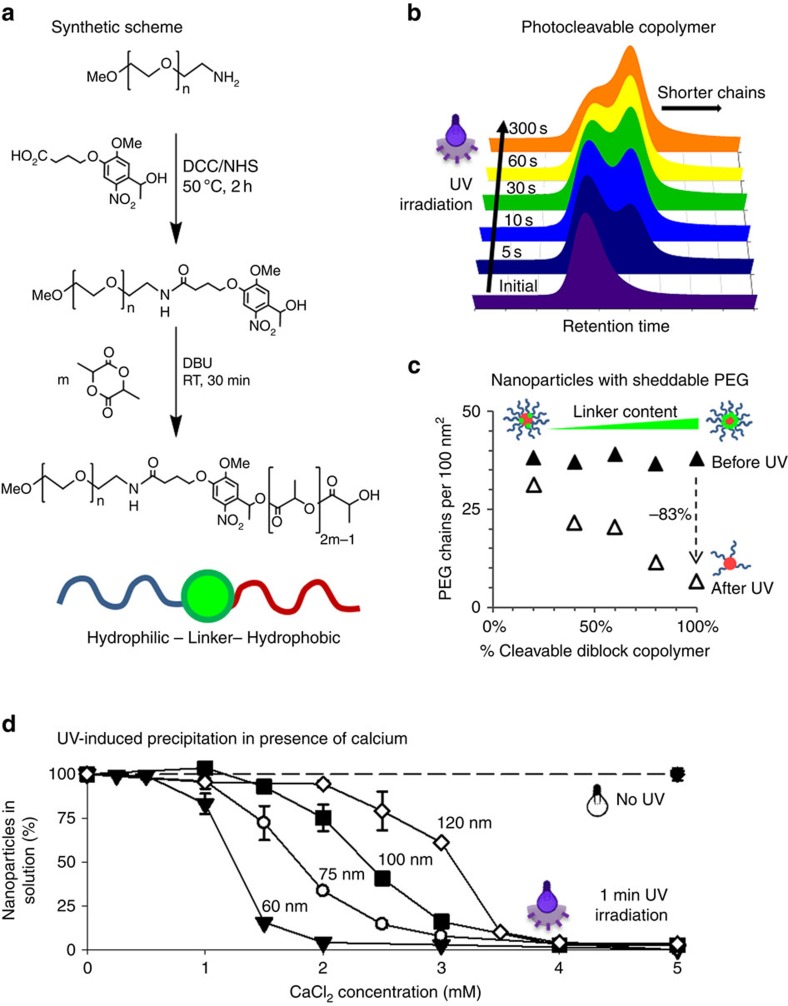
Photocleavable copolymers are used to prepare nanoparticles with inducible precipitation. (**a**) Photocleavable PEG-*b*-PLA copolymers can be synthesized using a photolabile linker. (**b**) The light-induced cleavage of the polymer into smaller chains can be followed by gel permeation chromatography. (**c**) Nanoparticles can be prepared using different ratios of photocleavable to non-cleavable copolymers. On ultraviolet irradiation, these core-shell nanoparticles lose their PEG corona proportionally to the content of photocleavable copolymer. (**d**) Nanoparticles prepared with photocleavable copolymer can be rapidly and efficiently precipitated in the presence of CaCl_2_ by ultraviolet irradiation. Values represent mean±s.d., *n*=3.

Photocleavable poly(ethylene glycol)-*b*-poly(lactic acid) (PEG-*b*-PLA) copolymers were synthesized in a two-step procedure. First, a hydroxyethyl photolinker was conjugated to *O*-(2-aminoethyl)*-O′*-methyl-PEG (5 kDa molecular mass). The obtained polymer was then used as a macroinitiator for the ring-opening polymerization of *meso*-lactide at room temperature; 1,8-diazabicyclo[5.4.0]undec-7-ene (DBU) served as a catalyst (Fig. 1a). The reaction yielded narrow-dispersed polymers with tunable PLA block length and molar masses between 5 and 35 kDa (Supplementary Table 1). The photocleavage of the synthesized polymers was investigated by gel permeation chromatography (GPC). Irradiation with ultraviolet light (320–395 nm, 10 mW cm^−2^) in water immediately triggered polymer degradation; however, photocleavage plateaued after ∼1 min at ∼70–80% cleavage (Fig. 1b and Supplementary Fig. 1). This can possibly be attributed to non-cleavable diblock copolymer resulting from the presence of unmodified methoxy-PEG or unreacted *O*-(2-aminoethyl)-*O′*-methyl-PEG, two impurities that can initiate the ring-opening polymerization of *meso*-lactide.

Using different ratios of photocleavable to non-cleavable diblock copolymer, we prepared nanoparticles with increasing levels of sheddable PEG (Fig. 1c and Supplementary Fig. 2a). The PEG density on the surface of the nanoparticles was quantified by ^1^H-NMR in deuterium oxide (D_2_O) and D_2_O/acetonitrile-d_3_ mixtures^15^. Data suggest that the initial density of the hydrophilic corona is not affected by the ratio of photocleavable to non-cleavable diblock copolymer (Fig. 1c and Supplementary Fig. 2a). Such nanoparticles can be prepared in a range of sizes; herein, particles with diameters of 60 nm (Supplementary Fig. 2a) and 90 nm (Fig. 1c) were prepared. After 1 min of irradiation with ultraviolet light, the PEG density decreased proportionally to the amount of photocleavable diblock copolymer in the nanoparticles. This confirms the photodegradability of the amphiphilic diblock copolymers, and their ability to form nanoparticles with sheddable PEG corona by self-assembly in water. Interestingly, these nanoparticles maintained colloidal stability in pure water after ultraviolet irradiation and PEG shedding, with only small alterations in their hydrodynamic diameter being detectable (Supplementary Fig. 2b). Given the strongly negative zeta potential of ultraviolet-irradiated nanoparticles (Supplementary Fig. 2c), it is conceivable that this preserved colloidal stability is in part due to electrostatic repulsion.

This assumption was further confirmed by the observation that neutralization of the surface charge, for example, by lowering the pH or by addition of cations, could trigger precipitation of ultraviolet-irradiated nanoparticles (Supplementary Fig. 2d). In the presence of 5 mM CaCl_2_, extensive precipitation was observed within seconds. To quantify the amount of nanoparticles remaining in suspension, nanoparticles with diameters of 60, 75, 100 and 120 nm were prepared using [^14^C]-labelled poly(lactic-*co*-glycolic acid) (PLGA). It was shown that >95% of the radiolabelled nanoparticles were efficiently removed by addition of CaCl_2_, followed by 5 min of very mild centrifugation (100 r.c.f.) and decantation; a filtration step was not required (Fig. 1d). Due to the colloidal stability of nanoparticles that have lost their PEG corona, the system acts as an ‘AND gate' where both stimuli (ultraviolet irradiation and neutralization of surface charges) are required to trigger precipitation. In agreement with the data obtained by GPC, irradiation times as short as 15 s could trigger precipitation in the presence of CaCl_2_ (Supplementary Fig. 3). Nonetheless, an irradiation time of 1 min was chosen to ensure optimal precipitation. Similarly, a CaCl_2_ concentration of 5 mM has been deemed sufficient to precipitate all nanoparticle formulations, although data suggest that smaller nanoparticles precipitate in the presence of lower CaCl_2_ concentrations, conceivably because of increased surface energy.

### Interactions between nanoparticles and chemicals

**Figure 2:**
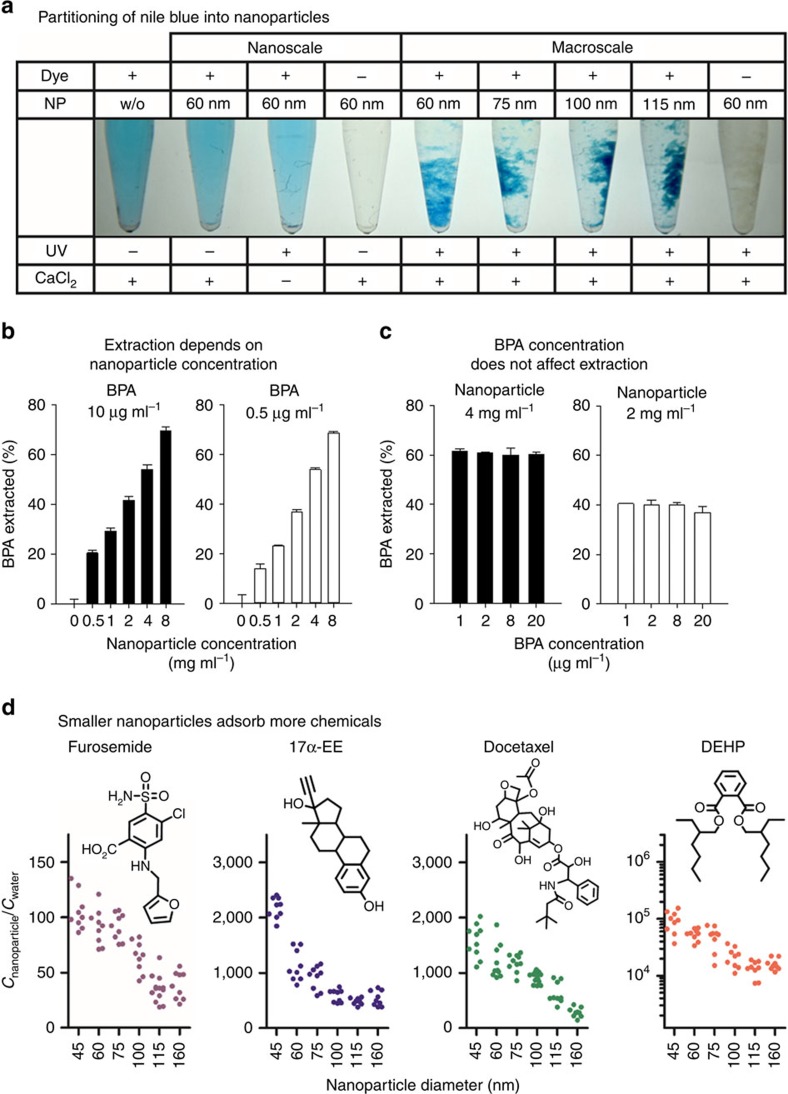
Phase separation highlights the adsorption of small molecules on the surface of the nanoparticles. (**a**) Precipitation of the nanoparticles shows that dye molecules concentrate preferentially on the nanoparticle surface, perceivably reducing the concentration in the supernatant. (**b**) The relative extraction of BPA is more efficient when the concentration of nanoparticles is increased. This phenomenon is observed at 10 μg ml^−1^ and 0.5 μg ml^−1^ of BPA. Values represent mean±s.d., *n*=3. (**c**) For concentrations<20 μg ml^−1^, when the nanoparticle concentration is kept constant, the extraction process remains unchanged irrespective of the amount of BPA. Values represent mean±s.d., *n*=3. (**d**) The adsorption onto the surface of the nanoparticles can be evidenced for chemicals with different chemical structures and physicochemical properties. Smaller nanoparticles appear to show higher adsorption than larger ones. Values represent each replicate, *n*=9–12.

The possibility to trigger rapid and efficient precipitation of different-sized nanoparticles allowed studying their interactions with small molecules. As a first proof-of-concept, irradiated and non-irradiated nanoparticles were incubated with aqueous solutions of amphiphilic dyes (Fig. 2a and Supplementary Fig. 4). On precipitation with CaCl_2_, partitioning of Nile blue and other dyes was observed, most likely due to interactions with the hydrophobic core of the nanoparticles. The nanoparticles were able to sequester sufficient amounts of these compounds to significantly reduce their concentration in the supernatant. Obviously, the phase separation resulting from the precipitation of the nanoparticles can be utilized to extract hydrophobic molecules from solution. The capability of extracting contaminants from water was then studied using bisphenol A (BPA) as a model compound (Fig. 2b,c). Both the concentration of BPA and the amount of nanoparticles were varied to study extraction at different nanoparticle-to-BPA ratios. Interestingly, only changing the amount of nanoparticles influenced the relative amounts of BPA that could be extracted from solution (percentage of initial BPA removed; Fig. 2b). For a given concentration of nanoparticles, the partitioning remained constant over a range of BPA concentrations (Fig. 2c). This suggests that, under these dilute conditions, the nanoparticles act as a sink for the small molecule. The amount of hydrophobic material, in which BPA is probably more soluble than in water, dictates the extent of interaction near equilibrium.

To better understand how various chemicals interact with the system, nanoparticles with diameters ranging from 45 to 160 nm were incubated with different molecules (Fig. 2d and Supplementary Fig. 5a). In general, after 1 h of incubation, nanoparticles with a diameter of 45 nm seemed to adsorb higher quantities of amphiphilic molecules than larger particles. Enhanced adsorption appears to correlate with the surface-to-volume ratio of nanoparticles (Supplementary Fig. 5b) and the partition coefficient (log *P*) of chemicals (Supplementary Fig. 6). This suggests that hydrophobic forces at the interface are at least partially responsible for the interactions between the nanomaterial and the small molecules. Although these findings are intuitive, to the best of our knowledge, it is the first time that the interactions of small molecules with pre-formed nanoparticles can be directly measured. Since phase separation occurs within seconds at room temperature and without any changes in the pH, we believe that the measured partitioning might offer a good representation of the interactions between small molecules and nanoparticles. As such, the phenomenon might very well have broader fundamental implications: from understanding the processes of drug encapsulation and release in drug delivery, to the phenomena involved in the self-assembly of ligand-functionalized nanoparticles^16^. Generally speaking, our observations could also offer new strategies for the design of nanoscale structures or provide tools for the separation or concentration of analytes. With this in mind, we, herein, exploited the adsorption of molecules on photo-responsive nanoparticles as a unique opportunity for the extraction of toxic chemicals found in the environment.

### Extraction and photodegradation of pollutants

**Figure 3:**
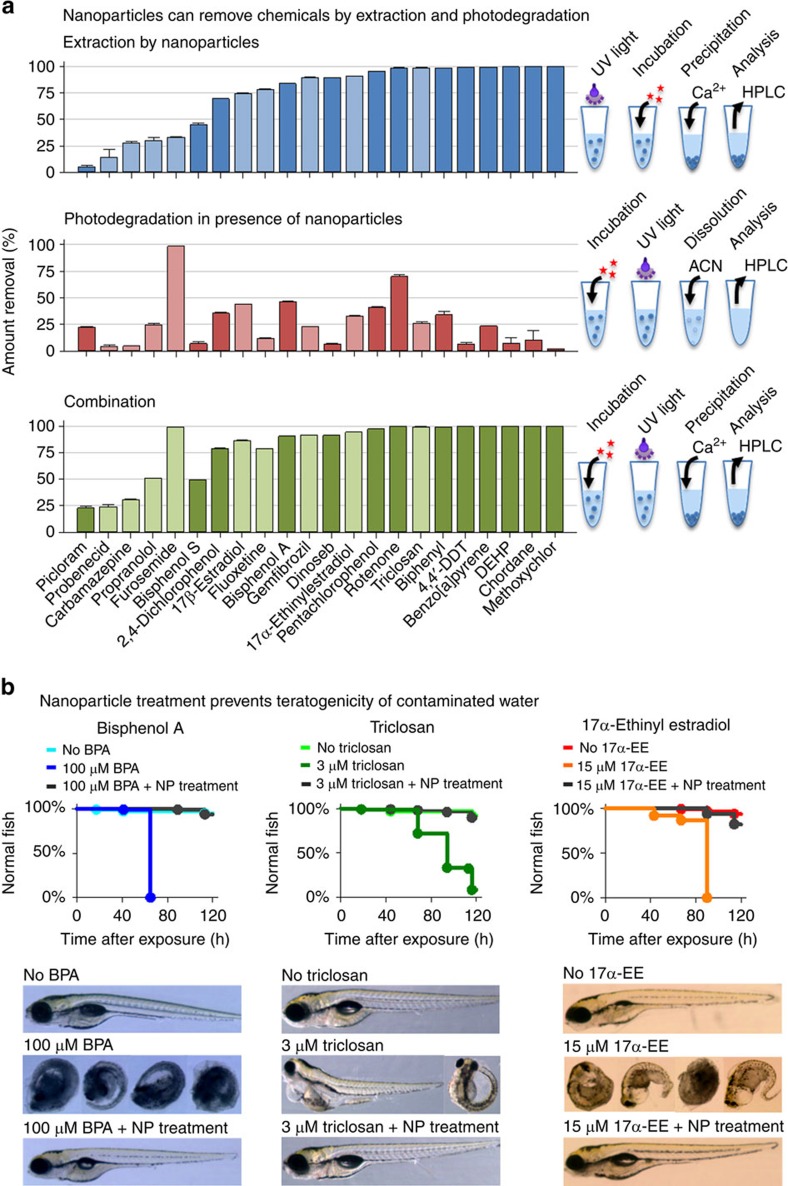
Extraction of chemicals by nanoparticles allows efficient removal of pollutants, mitigating the toxicity of contaminated water. (**a**) Small quantities of photo-responsive nanoparticles can remove various chemicals from water by extraction, photodegradation or a combination of both principles. Depending on the hydrophobicity, the nanoparticles can extract 5–100% of the chemicals, while the extent of photodegradation remains <50% for most chemicals. Together, the two processes can remove 25–100% of the chemicals from solution. Values represent mean±s.d., *n*=3. (**b**) In zebrafish embryos, treatment of water with photo-responsive nanoparticles mitigates the teratogenicity of BPA, 17α-EE and triclosan (log rank, *P*<0.001, *n*=96).

The ability of nanoparticles to extract different types of chemicals was investigated. The interactions between nanoparticles (60 nm diameter) and 22 chemicals covering a broad range of structures and hydrophobicity were studied (Fig. 3a). These molecules were chosen because they were endocrine disruptors, biopersistent molecules or pharmaceuticals that cause deleterious effect in humans or other species. Figure 3a shows that one single extraction step with <1% (weight per volume) of nanoparticles is sufficient to remove between 5 and 100% of the added chemicals, depending on their hydrophobicity. Since the removal of nanoparticles necessitates ultraviolet irradiation, photodegradation of the chemicals was also evaluated in a separate experiment. For this, the nanoparticles were incubated with the chemicals and irradiated without phase separation (that is, in the absence of CaCl_2_). Under these conditions, most chemicals were degraded only to a minor extent (that is, <50%), while other substances (for example, furosemide and rotenone) were more prone to photodegradation (Fig. 3a). In general, the presence of the photolinker appeared to contribute to the degradation reaction since irradiation of the chemicals in aqueous solution without the linker resulted in much less photodegradation (Supplementary Fig. 7). On this linker, the photolysis of *o-*nitrobenzyl alcohol derivatives occurs in a Norrish-type II reaction, which involves a highly reactive biradical intermediate^17^. Combined with ultraviolet irradiation, this may lead to photo-oxidation of susceptible chemicals^18^,^19^,^20^. Photodegradation might be further enhanced if the chemicals are adsorbed on the surface of nanoparticles and in close proximity to the generated biradicals. From the perspective of environmental remediation, photodegradation might be beneficial as it offers an alternative route for the destruction of harmful chemicals. However, it is important to note that the process of photodegradation might prompt the generation of even more toxic molecules (for example, triclosan, see ref. 21). The combination of two concomitant principles of removing chemicals, namely sequestration by non-specific adsorption and photocatalytic degradation, could possibly ensure that toxic byproducts are nonetheless eliminated. Together, the process of extraction and photodegradation provides efficient removal of most chemicals from water; <1% (weight per volume) of nanoparticles is sufficient to remove 25–100% of the added chemicals (Fig. 3a).

### Teratogenicity studies in zebrafish embryos

To measure the impact of extraction and partial degradation on the toxicological profile of chemicals, a zebrafish embryo teratogenicity experiment was carried out. While the used concentrations (100, 3 and 15 μM) of BPA, triclosan and 17α-ethinyl estradiol (17α-EE) were deliberately above the teratogenic concentrations of the compounds^22^,^23^, Fig. 3b shows that treatment of water with nanoparticles significantly reduced the teratogenicity of the three studied chemicals. For comparison, the use of non-photocleavable nanoparticles or ultraviolet irradiation without any nanoparticles failed to mitigate the toxicity of BPA (Supplementary Fig. 8a). Embryo teratogenicity studies also showed that functionalization of PEG with the hydroxyethyl photolinker did not compromise the innocuousness of the polymer, both before and after ultraviolet irradiation (Supplementary Fig. 8b). While this *in vivo* experiment has limitations, it suggests that treatment of contaminated water with photo-responsive nanoparticles might prove advantageous to decrease the toxic effects of some chemicals. In agreement with the data presented in Fig. 3a, the *in vivo* experiment corroborates that small quantities of photo-responsive nanoparticles are able to remove large amounts of toxic molecules from water, without generating obviously toxic byproducts. Together, this data confirms the potential of the developed platform for applications in water purification.

### Demonstration of potential applications in pilot studies

**Figure 4:**
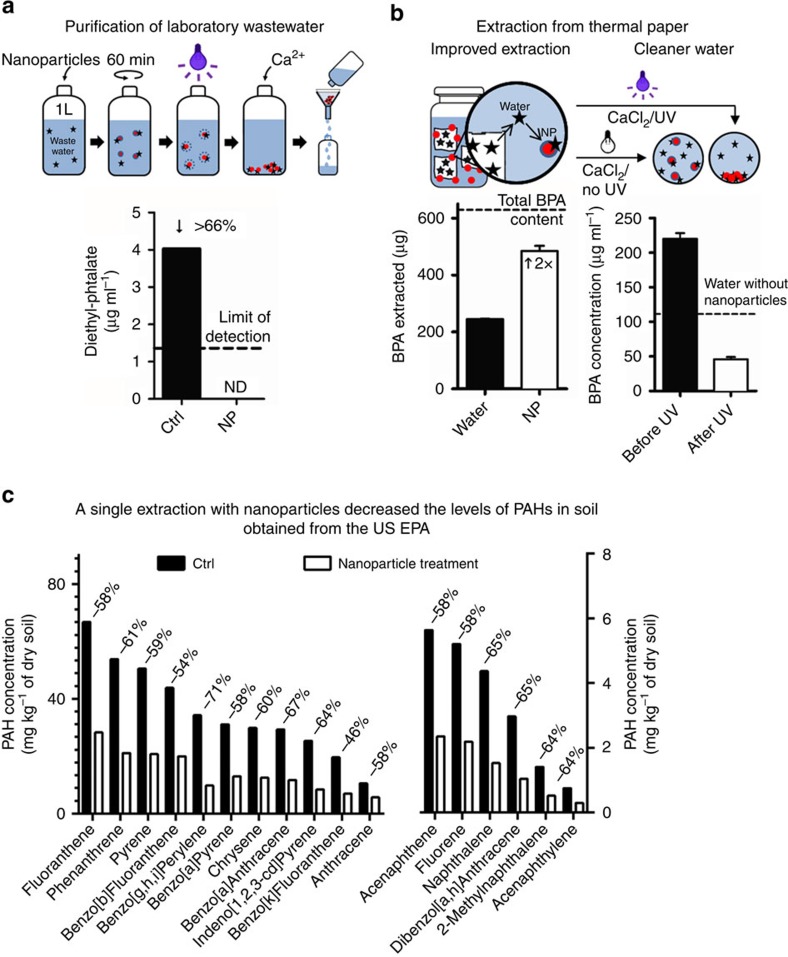
Pilot studies on laboratory wastewater, thermal printing paper and contaminated soil emphasize the feasibility of the approach. (**a**) Using photo-responsive nanoparticles, wastewater can be purified from all detectable traces of contaminants. (**b**) The BPA present in thermal paper pulp can be extracted more efficiently using nanoparticles. Furthermore, the process water can be purified to contain lower amounts of residual BPA. (**c**) PAHs can also be efficiently extracted from soil using nanoparticles, affording cleaner soil.

In the interest of demonstrating potential applications of the technology in small-scale pilot studies, contaminated water, thermal printing paper and soil samples were treated with photo-responsive nanoparticles. Samples of laboratory wastewater from various MIT buildings were collected by the Institute's Environment, Health and Safety (EHS) Office. It is important to note that the measured phthalate concentrations were much below the reporting value set by the Massachusetts Water Resources Authority (MWRA). Nevertheless, the treatment with photo-responsive nanoparticles proved to be effective in decontaminating the sample; as little as 750 mg of nanoparticles were required to remove all detectable traces of phthalates from 1 litre of water (Fig. 4a).

In the second example, nanoparticles were used to extract BPA from commercially available thermal printing paper, which contains large concentrations of the endocrine disrupting compound and is considered a major source of human BPA exposure^24^. Treating the sample with 1% (weight per volume) of nanoparticles was almost twice as effective as extraction with an equivalent volume of water (that is, 77% of the total BPA content was removed versus 39%) (Fig. 4b). This suggests that nanoparticles can create a sink for hydrophobic molecules and facilitate desorption from the solid matrix. Furthermore, photo-responsive nanoparticles allow removal of >75% of the BPA present in the resulting process water (Fig. 4b). Although intended as an example, this pilot experiment also hints at potential applications of photo-responsive nanoparticles to reduce the discharge of chemicals from industrial processes. For example, it has been estimated by the European Institute for Health and Consumer Protection that ∼700 metric tons of BPA will end up in paper recycling sites each year^25^. The extraction and concentration of BPA by using photo-responsive nanoparticles might, therefore, decrease the residual BPA in the recovered paper and provide an efficient way of reducing environmental emissions from paper recycling.

Finally, the feasibility of decontaminating soil samples was investigated. The US Environmental Protection Agency kindly provided a soil sample contaminated with various amounts of polycyclic aromatic hydrocarbons (PAHs). Because of the volatility of some PAHs, the effectiveness of the extraction procedure was compared with a control group, which was treated according to the same protocol with an equivalent volume of water. A single extraction using <0.5% (weight per volume) of nanoparticles could significantly reduce the concentrations of all determined PAHs. The PAH levels measured in the treated sample were 46 to 71% lower than in the control group (Fig. 4c). In agreement with the results obtained with BPA-containing thermal paper, this suggests that nanoparticles can facilitate desorption and sequestration of hydrophobic molecules from soil particles. Moreover, the extraction procedure appeared to remain effective although the sample contained a variety of structurally distinct chemical entities (17 different PAHs were measured). Apparently, the decontamination process is mostly unaffected by the presence of multiple competing compounds.

## Discussion

In this work, we proposed to harness the properties of polymeric nanoparticles, such as high surface-to-volume ratio, colloidal stability, biodegradability and the ability to respond to external stimuli, to benefit the field of environmental remediation. The unique design of photo-responsive nanoparticles enables their permanent conversion to macroscopic aggregates and facilitates their removal from the treated sample. In other words, it allows using self-assembled nanostructures without significant environmental exposure to nanomaterials. Second, it allows the concentration of contaminants to a smaller volume, which can be treated more easily in downstream processes. To further reduce potential risks associated with the use of nanomaterials in the environment, the nanoparticles are prepared from biodegradable polymer precursors. Certain polymers, such as PEG, PLA or PLGA, are generally recognized as safe for use in cells, animals and humans. They are used as food additives as well as excipients in various drugs and cosmetics approved by the US Food and Drug Administration. Nano and microparticles prepared from these polymers have been utilized in biomedical applications for >20 years^26^,^27^. Moreover, recent developments in the industrial production of PLA from sustainable sources have made the synthesis of the polymer more economical and environment friendly^28^. Altogether, the proven safety of the material, as well as the innovative design allowing its easy separation may provide an innocuous alternative to the use of nanotechnology in environmental remediation.

In summary, the proof-of-concept presented here illustrates the potential of photo-responsive nanoparticles for chemical extraction and environmental remediation. In its current configuration, the platform presents the drawback of releasing non-biodegradable PEG chains in the environment. While PEG is considered a safe food additive (FDA Code of Federal Regulations 21 CFR 172.820) and its functionalization with the hydroxyethyl photolinker did not cause obvious toxicity in zebrafish embryo teratogenicity studies, it is conceivable to stabilize future generations of the system with biodegradable polysaccharides or polypeptides. Utilizing light to trigger nanoparticle precipitation appears advantageous, because ultraviolet irradiation is already used to control bacterial growth in water filtration systems. Nanoparticles with photoinduced precipitation could, therefore, be implemented in the existing infrastructure, for example, after chemical spills. Nonetheless, the nanoparticle platform is easily amenable to other architectures that could render the precipitation ‘switch' dependent on temperature, ionic strength or pH^29^. These alternative stimuli might be particularly appealing to allow the design of reusable particles, or when photodegradation of the adsorbed molecules would be deleterious. For example, extraction with nanoparticles could be used in analytical or diagnostic applications to concentrate analytes from blood or urine before quantification. Similarly, such nanoparticles, which are biodegradable, safe and easy to retrieve, could be applied in the food industry, when mild extraction procedures are needed to preserve nutrients or flavour^30^. Stimuli-responsive nanoparticles could also be used in agricultural production to control the diffusion and movement of pesticides or herbicides in agricultural soils^31^.

More generally, the rapid phase separation allows measuring the partitioning of small molecules between the aqueous phase and nanoparticles. Data suggests that for many chemicals, interactions are enhanced with decreasing particle size. We believe that these findings will contribute to a better understanding of adsorption phenomena occurring at the interface of nanomaterials, and possibly have broad implications in various fields: from analytical, technical or industrial applications, such as those mentioned above, to more fundamental concerns, such as understanding how nanomaterials interact with their environment.

## Methods

### Synthesis of photocleavable PEG-*b*-PLA copolymers

Photocleavable PEG-*b*-PLA diblock copolymers were synthesized from *O*-(2-aminoethyl)-*O′*-methylpolyethylene glycol, molecular mass 5 kDa. In the first step, Novabiochem hydroxyethyl photolinker (1.23 mmol, 368 mg), *N*-hydroxysuccinimide (NHS, 1.23 mmol, 142 mg) and *N,N′*-dicyclohexylcarbodiimide (DCC, 1.23 mmol, 254 mg) were dissolved in 10 ml of anhydrous 1,4-dioxane. The reaction mixture was stirred for 6 h at room temperature under light protection. The precipitated dicyclohexylurea was filtered off, and the filtrate was combined with a solution of *O*-(2-aminoethyl)-*O′*-methylpolyethylene glycol (0.82 mmol, 4.0 g) and sodium bicarbonate (1.23 mmol, 84 mg) in 10 ml of water. The reaction mixture was stirred over night at 50 °C under light protection. The next day, the solvent was evaporated and the residue was taken up in water. The raw product was extracted with dichloromethane (DCM). The combined organic phases were dried over anhydrous sodium sulfate, filtered and concentrated. The product was crystallized at 0 °C under vigorous stirring by dropwise addition of diethyl ether. The faint yellow precipitate was collected by filtration, washed with cold diethyl ether and dried under vacuum to yield 4.0 g (95.7%) of polymer. ^1^H-NMR (CDCl_3_, 300 MHz): δ 1.51 p.p.m. (d, 3H, −CH(C*H*_*3*_)OH), 2.18 p.p.m. (m, 2H, −C(O)CH_2_C*H*_*2*_CH_2_−), 2.40 p.p.m. (t, 2H, −C(O)C*H*_*2*_CH_2_CH_2_−), 3.36 p.p.m. (s, 3H, *H*_*3*_COCH_2_CH_2_−), 3.62 p.p.m. (s, 444H, −OC*H*_*2*_C*H*_*2*_−), 3.96 p.p.m. (s, 3H, *H*_*3*_CO−), 4.09 p.p.m. (t, 2H, −C(O)CH_2_CH_2_C*H*_*2*_−), 5.52 p.p.m. (q, 1H, −C*H*(CH_3_)OH), 7.31 p.p.m. (s, 1H, ar), 7.54 p.p.m. (s, 1H, ar)

The obtained polymer was used as macroinitiator for the ring-opening polymerization of 3,6-dimethyl-1,4-dioxane-2,5-dione. PEG-*b*-PLA diblock copolymers with different PLA block lengths were synthesized (Supplementary Table 1). In a typical polymerization, the macroinitiator (0.08 mmol, 0.40 g) was dissolved in 10 ml of anhydrous DCM; 3,6-dimethyl-1,4-dioxane-2,5-dione (11.09 mmol, 1.60 g) and DBU (0.11 mmol, 106 μl) were added. The round-bottom flask was sealed, and the reaction mixture was stirred at room temperature. After 1 h, the polymerization was quenched with benzoic acid (0.55 mmol, 67 mg). The viscous solution was added to vigorously stirred diethyl ether. The white solid was isolated and dried under vacuum at 45 °C to yield 1.4 g (67.5%) of PEG5k-*b*-PLA20k diblock copolymer. ^1^H-NMR (CDCl_3_, 300 MHz): δ 1.56 p.p.m. (844H, −C(C*H*_*3*_)H−), 3.37 p.p.m. (3H, −*H*_*3*_COCH_2_CH_2_−), 3.64 p.p.m. (444H, −OC*H*_*2*_C*H*_*2*_−), 5.16 p.p.m. (278H, −C(CH_3_)*H*−).

### Preparation of photo-responsive nanoparticles

Nanoparticles were prepared by a nanoprecipitation method. To this end, different amounts of photocleavable PEG-*b*-PLA, non-cleavable PEG-*b*-PLA, PLA or PLGA were dissolved in acetonitrile at 10 mg ml^−1^ (Supplementary Table 2). To obtain nanoparticles of different sizes, the ratio of PEG-*b*-PLA to PLA or PLGA was varied as listed in Supplementary Table 2. For the preparation of radiolabelled nanoparticles, [^14^C]-labelled PLGA (Moravek Biochemicals, CA, custom synthesis) was integrated in the polymer solution. Then, 1 ml of the polymer solution was dropped into 10 ml of water under magnetic stirring. The nanoparticle suspension was concentrated by ultrafiltration and washed at least three times with water. The size of the nanoparticles (Z-average size and polydispersity index) was measured by dynamic light scattering in water at 22 °C with a 173° backscatter angle, using a Malvern Zetasizer Nano ZS (Malvern Instruments, Westborough, MA). The concentration of nanoparticles in each formulation was measured in duplicates by gravimetric analysis after lyophilization.

### Determination of the PEG density

The density of PEG on the surface of the nanoparticles before and after ultraviolet irradiation was measured by ^1^H-NMR^15^. In a typical experiment, nanoparticles were irradiated with ultraviolet light for 1 min (320–395 nm, 10 mW cm^−2^, DYMAX BlueWave 200 UV Curing Spot Lamp, Dymax Corporation, Torrington, CT), transferred into an ultrafiltration device (molecular weight cutoff 50 or 100 kDa), and washed four times with D_2_O to remove residual water and cleaved PEG chains. After solvent exchange, 1% (weight per volume) of deuterated trimethylsilyl propanoic acid (TMSP) was added as an internal standard. ^1^H-NMR spectra were recorded on a Bruker 400 MHz spectrometer (Bruker, Billerica, MA). The ratio of the integral of the methylene protons of PEG (3.6 p.p.m.) to the integral of the methyl protons of TMSP (0 p.p.m.) was then compared with a calibration curve of PEG in D_2_O to calculate the concentration of hydrated PEG in the sample. To assess the total polymer concentration in the sample, the nanoparticles were dissolved by adding an equal volume of acetonitrile-d_3_. The ratio of the integral of the methyl protons of lactic acid (1.9 p.p.m.) to the integral of the methyl protons of TMSP (0 p.p.m.) was then compared with a calibration curve of the copolymer in D_2_O/acetonitrile-d_3_ to calculate the concentration. The density of the PEG corona (PEG chains per 100 nm^2^) was obtained by relating the concentration of PEG (in percentage by mass, wt % PEG) in the nanoparticle (that is, concentration of hydrated PEG/total polymer concentration) to the surface area (*S*) and nanoparticle volume (*V*) using a density (*ρ*) of 1.2 g cm^−3^:


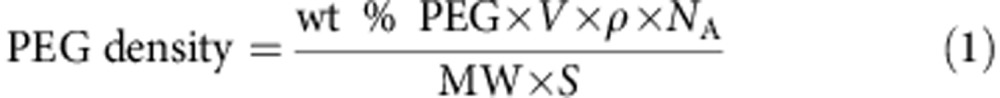


where *S* and *V* are calculated using the Z-average diameter of the nanoparticle distribution (in centimetre), MW is the PEG chain average molecular weight, and *N*_A_ is Avogadro's number.

### Precipitation of photo-responsive nanoparticles

To study the precipitation of nanoparticles after ultraviolet irradiation and in the presence of CaCl_2_, [^14^C]-labelled nanoparticles of different sizes were prepared as described above. Tenfold concentrated stock solutions of CaCl_2_ were prepared; 8 μl of these solutions were added to 72 μl of nanoparticles to give final CaCl_2_ concentrations ranging from 0 to 5 mM. The samples were irradiated with ultraviolet light for 0–5 min (320–395 nm, 10 mW cm^−2^, DYMAX BlueWave 200 UV Curing Spot Lamp). When studying the impact of different CaCl_2_ concentrations, the irradiation time was fixed at 1 min; when evaluating the impact of ultraviolet irradiation time, the CaCl_2_ concentration was fixed at 5 mM. The irradiated samples were then centrifuged for 5 min at 100 r.c.f.; then, 20 μl of the supernatant were analysed by scintillation counting on a Tri-Carb 2810 TR Liquid Scintillation Analyzer (Perkin Elmer, Waltham, MA). The amount of particles remaining in suspension was measured by comparing the measured radioactivity to that in untreated samples. Each experiment was conducted in triplicate.

### Interactions between nanoparticles and chemicals

The interactions between nanoparticles and various chemicals were assessed by measuring their concentrations before and after extraction with three different concentrations of nanoparticles. Prior to the experiment, nanoparticles were irradiated with ultraviolet light for 1 min (320–395 nm, 10 mW cm^−2^, DYMAX BlueWave 200 UV Curing Spot Lamp). A stock solution of the tested chemical was prepared in ethanol (concentration 1 mg ml^−1^); the stock solution was diluted with water and ethanol to 20 μg ml^−1^ (10% ethanol content). In a polypropylene microcentrifuge tube, 10 μl of this solution were added to various amounts of ultraviolet-irradiated nanoparticles and water to give a final volume of 100 μl. These tubes were incubated under horizontal shaking for 1 h, supplemented with 10 μl of 55 mM CaCl_2_ and centrifuged for 5 min at 100 r.c.f. Subsequently, 50–60 μl of the supernatant were carefully removed and analysed by high-performance liquid chromatography (HPLC) according to the procedures described in the Supplementary Methods. In parallel, a control was conducted for each chemical, following the same procedure but using non-ultraviolet-irradiated nanoparticles. These particles were diluted with acetonitrile (1:1) before HPLC analysis. The partition coefficient was measured by comparing the amount of chemical remaining in the water phase according to equation (2):


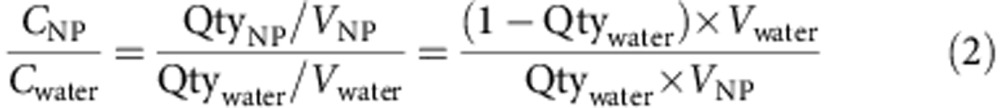


where Qty_NP_ and Qty_water_ are the quantities of chemical present on the nanoparticles and in the water phase after extraction, respectively; *V*_NP_ and *V*_water_ are the volumes of nanoparticles and water, respectively. *V*_NP_ was calculated by dividing the nanoparticle concentration by a density of 1.2 g ml^−1^, and *V*_water_ was calculated by subtracting *V*_NP_ from the total volume (0.11 ml). Each experiment was conducted using at least three replicates of three different nanoparticle concentrations.

### Extraction and photodegradation of pollutants

Stock solutions of the tested chemicals were prepared in ethanol (concentration 1 mg ml^−1^); the stock solutions were diluted with water and ethanol to 20 μg ml^−1^ (10% ethanol content). To quantify the removal of chemicals by extraction, photodegradation and the combination of both, 80 μl of the nanoparticle dispersion (initial concentration 8 mg ml^−1^) were mixed with 10 μl of the diluted solution of the chemical (nanoparticle concentration 7.1 mg ml^−1^, concentration of the chemical 2.2 μg ml^−1^). For each experiment, the addition of the chemical and the irradiation of the sample with ultraviolet light (320–395 nm, 60 s at 10 mW cm^−2^, DYMAX BlueWave 200 UV Curing Spot Lamp) occurred in the order specified in Fig. 3a. After 1 h of incubation at room temperature on an orbital shaker, 10 μl of a solution of CaCl_2_ in water (concentration 50 mM) were added to precipitate the nanoparticles. In the photodegradation study, water was added instead to maintain the nanoparticles in suspension. The samples were vortexed and centrifuged for 5 min at 100 r.c.f. Afterwards, 30 μl of the clear supernatant were carefully removed and diluted with 30 μl of acetonitrile. The amount of the remaining chemical was determined by HPLC (see Supplementary Methods for experimental details).

### Teratogenicity studies in zebrafish embryos

All animal experiments were conducted using institutionally approved protocols (IACUC). In this study, the teratogenicity of solutions containing BPA, triclosan or 17α-EE was evaluated before and after treatment with 0.8% (weight per volume) of photo-responsive nanoparticles; nanoparticles with a diameter of 60 nm were chosen. After 1 h of incubation, the samples were irradiated with ultraviolet light for 1 min (320–395 nm, 10 mW cm^−2^, DYMAX BlueWave 200 UV Curing Spot Lamp); 55 mM CaCl_2_ was added to give a final CaCl_2_ concentration of 5 mM. The samples were centrifuged for 10 min at 1,000 r.c.f. to remove the precipitated nanoparticles; the clear supernatant was collected and diluted fivefold with makeshift aquarium water to final concentrations of 100 μM, 3 μM and 15 μM for BPA, triclosan and 17α-EE, respectively. Fertilized zebrafish embryos (strain T/AB 14) were plated in a 48-well plate (1 egg per well) and exposed to aquarium water (control group), treated and non-treated samples, beginning 8–10 h post fertilization. Embryos were housed in incubators at 28 °C and observed daily for death or major abnormalities (yolk sac oedema, pericardial oedema, axis curvature or delayed hatching). Larvae were imaged in aquarium water containing 3% carboxymethylcellulose and euthanized 120 h after exposure to chemicals. Each group contained 96 embryos to properly highlight differences in viability between treated, non-treated and control groups.

### Extraction of phthalates from wastewater

With the help of the Institute's EHS officers, wastewater was sampled in May 2014 from a post neutralization tank in MIT building 16 via a non-regulated MWRA port. The collected water was separated in two 1 l ultraclean glass bottles (obtained from GeoLabs, Inc., Analytical Laboratories, Braintree, MA) and kept at 4 °C until treatment and analysis. For water treatment, 750 mg of photo-responsive nanoparticles were suspended in 35 ml of water and added to 1 litre of wastewater; the sample was stirred at room temperature for 1 h. The stirred sample was then irradiated with ultraviolet light (320–395 nm) for 20 min at a light intensity of 10 mW cm^−2^ (DYMAX BlueWave 200 UV Curing Spot Lamp); 555 mg of CaCl_2_ were added to precipitate the nanoparticles. The precipitated nanoparticles were separated by filtration using a paper filter. A control, which contained an equal volume of water instead of nanoparticles, was treated following the same procedure. A single-blind water analysis was conducted on both samples according to standard operating procedures described in US EPA Method 625 by GeoLabs, Inc., Analytical Laboratories. The comparison presented in Fig. 4 shows the differences in concentration between the control and the treated sample.

### Extraction of BPA from thermal printing paper

Thermal printing paper was cut into small pieces of ∼0.5 × 0.5 cm. To estimate the total amount of BPA, 50 mg of paper were extracted with 1,000 μl of acetonitrile according to the following procedure. The samples were incubated at room temperature on an orbital shaker. After 1 h, the samples were filtered (1.2 μm pore size); 100 μl were removed and diluted with 10 μl of acetonitrile. The samples were vortexed and centrifuged for 5 min at 100 r.c.f. Afterwards, 30 μl were removed and diluted with 30 μl of acetonitrile. The amount of BPA was determined by HPLC using a calibration curve (see Supplementary Methods for experimental details). In a control experiment, 50 mg of paper were extracted with 1,000 μl of water. The samples were incubated and filtered as described above. Afterwards, 100 μl of the filtrate were diluted with 10 μl of water. The samples were vortexed and centrifuged for 5 min at 100 r.c.f.; 30 μl were removed and diluted with 30 μl of acetonitrile. The concentration of BPA was determined as described above. In the third group, 50 mg of paper were extracted with 1,000 μl of a dispersion of nanoparticles in water (concentration 8 mg ml^−1^). The samples were incubated and filtered as described above. Afterwards, 100 μl of the filtrate were diluted with 10 μl of a solution of CaCl_2_ in water (concentration 50 mM). To demonstrate the possibility of removing BPA from the aqueous phase, another 100 μl of the filtrate were irradiated with ultraviolet light for 1 min (320–395 nm, 10 mW cm^−2^, DYMAX BlueWave 200 UV Curing Spot Lamp) and diluted with 10 μl of a solution of CaCl_2_ in water (concentration 50 mM) to precipitate the nanoparticles. The samples were vortexed and centrifuged for 5 min at 100 r.c.f.; 30 μl of the clear supernatant were carefully removed and diluted with 30 μl of acetonitrile. The concentration of BPA was determined as described above. To quantify the amount of BPA remaining in the paper, the medium from the first extraction step was completely removed. Afterwards, all samples were extracted with 1,000 μl of acetonitrile and treated as described above. The results are presented as mean±s.d. based on the results of *n*=3 samples.

### Extraction of PAHs from soil

PAH-contaminated soil was generously donated by the US Environment Protection Agency. Seventy-five grams of soil was incubated with 430 mg of nanoparticles suspended in 35 ml of water. After 1 h of incubation, the samples were transferred into 50 ml polypropylene tubes and centrifuged at 10,000 r.c.f. for 15 min. The supernatant containing the nanoparticles was collected, and the soil was sent for analysis. A control was treated following the same procedure by using an equal volume of water instead of nanoparticles. A single-blind soil analysis was conducted on both samples according to standard operating procedures described in US EPA Method PAH SW8270C by GeoLabs, Inc., Analytical Laboratories. This laboratory is certified for environmental analysis by various authorities, including the Massachusetts Department of Environmental Protection (M-MA015).

## Additional information

**How to cite this article:** Brandl, F. *et al.* Nanoparticles with photoinduced precipitation for the extraction of pollutants from water and soil. *Nat. Commun.* 6:7765 doi: 10.1038/ncomms8765 (2015).

## Supplementary Material

Supplementary Information Supplementary Figures 1-9, Supplementary Tables 1-3 and Supplementary Methods
