# Supplementary material for: Nanoparticles with photoinduced precipitation for the extraction of pollutants from water and soil
Source: Nat Commun. 2015 Jul 21;6:7765. doi: 10.1038/ncomms8765 (PMC4518270; doi:10.1038/ncomms8765)
Supplement: Supplementary Information — Supplementary Figures 1-9, Supplementary Tables 1-3 and Supplementary Methods [file ncomms8765-s1.pdf]

SUPPLEMENTARY FIGURES

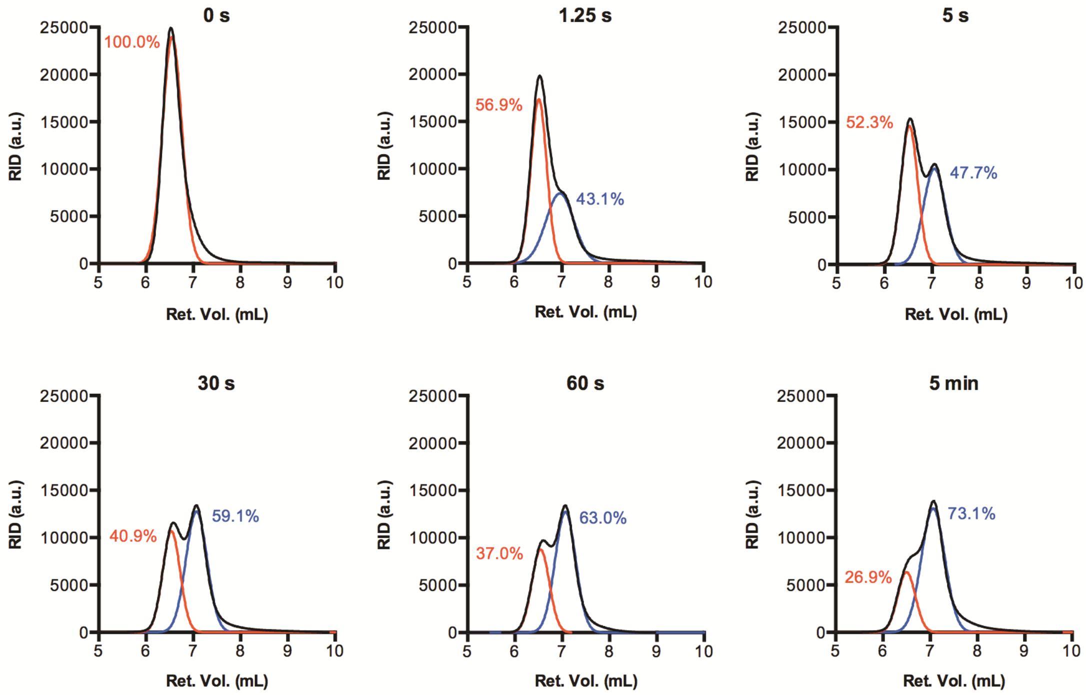

Supplementary Figure 1. Gel permeation chromatograms of a cleavable diblock copolymer before and after UV irradiation. Multi-peak-fitting analysis was performed to estimate the percentage of cleavage. After very short irradiation times (1.25–5 s), the peak of the larger PEG-*b*-PLA (in red) starts to decrease while the peak of the shorter PEG (in blue) starts to increase. Photocleavage appears to reach a plateau after ~60 s of irradiation.

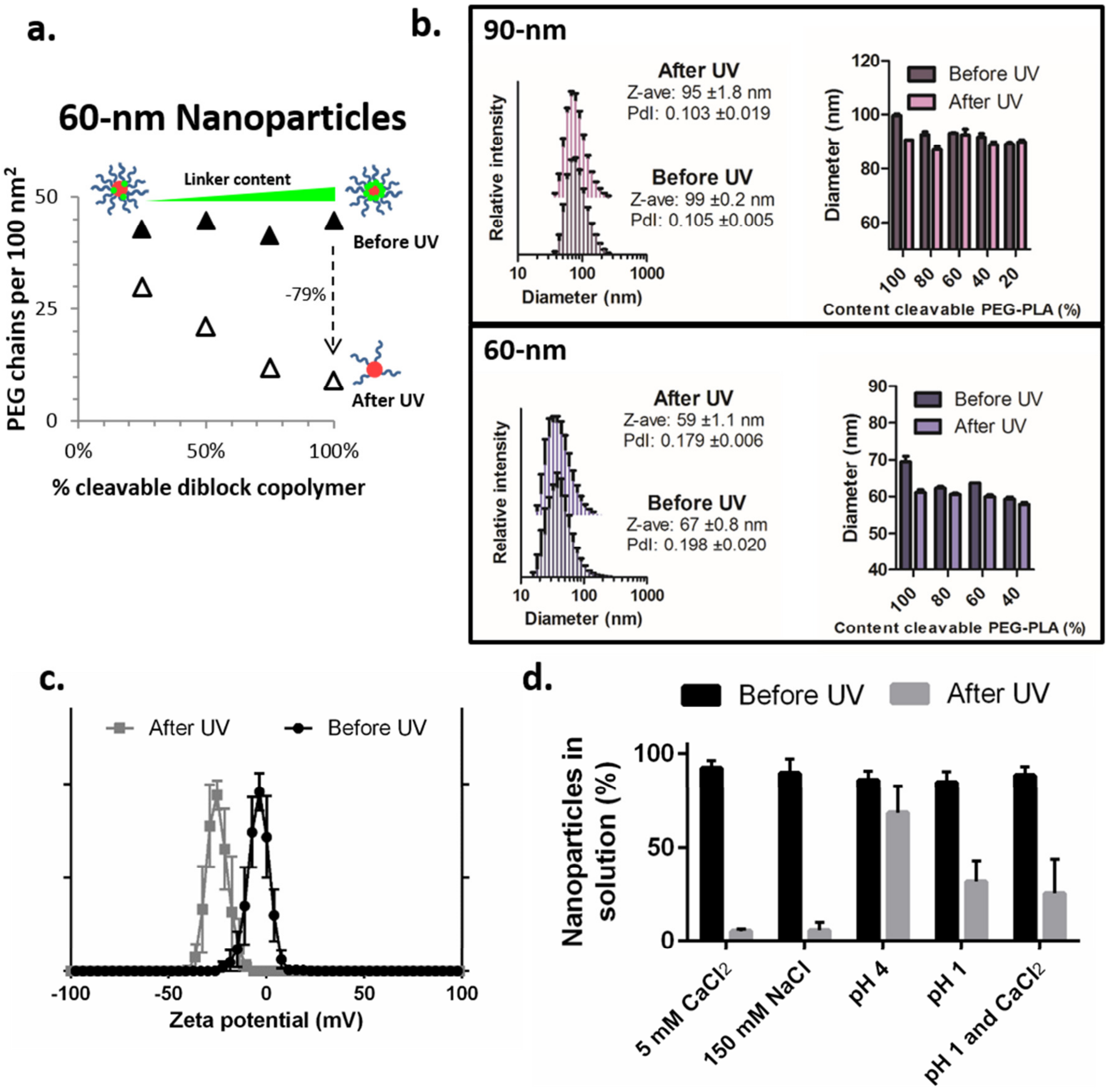

Supplementary Figure 2. **A.** 60-nm nanoparticles with different content of cleavable diblock copolymer can be synthesized. After synthesis, all nanoparticles have the same overall PEG density (full triangles). Upon UV irradiation, the PEG density on the surface of the nanoparticles (empty triangles) is inversely proportional to the amount of cleavable diblock copolymer used in the preparation of the nanoparticles. **B.** Shedding of the PEG corona does not appear to significantly affect the size distribution of the nanoparticles, possibly because the negative surface charges (COO^-) cause electrostatic repulsion. **C.** The shedding of the PEG corona increases the negative charge of the nanoparticles. **D.** Neutralization of the surface charge by cations (Ca^{2+} or Na^+) or acidic pH (pH 1) triggers precipitation of the nanoparticles.

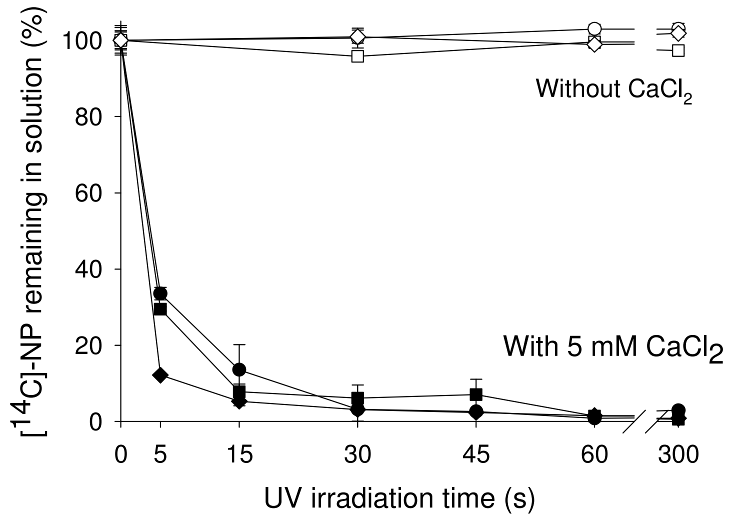

Supplementary Figure 3. After very short UV irradiation, the nanoparticles lose their protective PEG layer and precipitate in presence of calcium. The time needed to precipitate the nanoparticles is in agreement with the kinetics of copolymer cleavage obtained from GPC data.

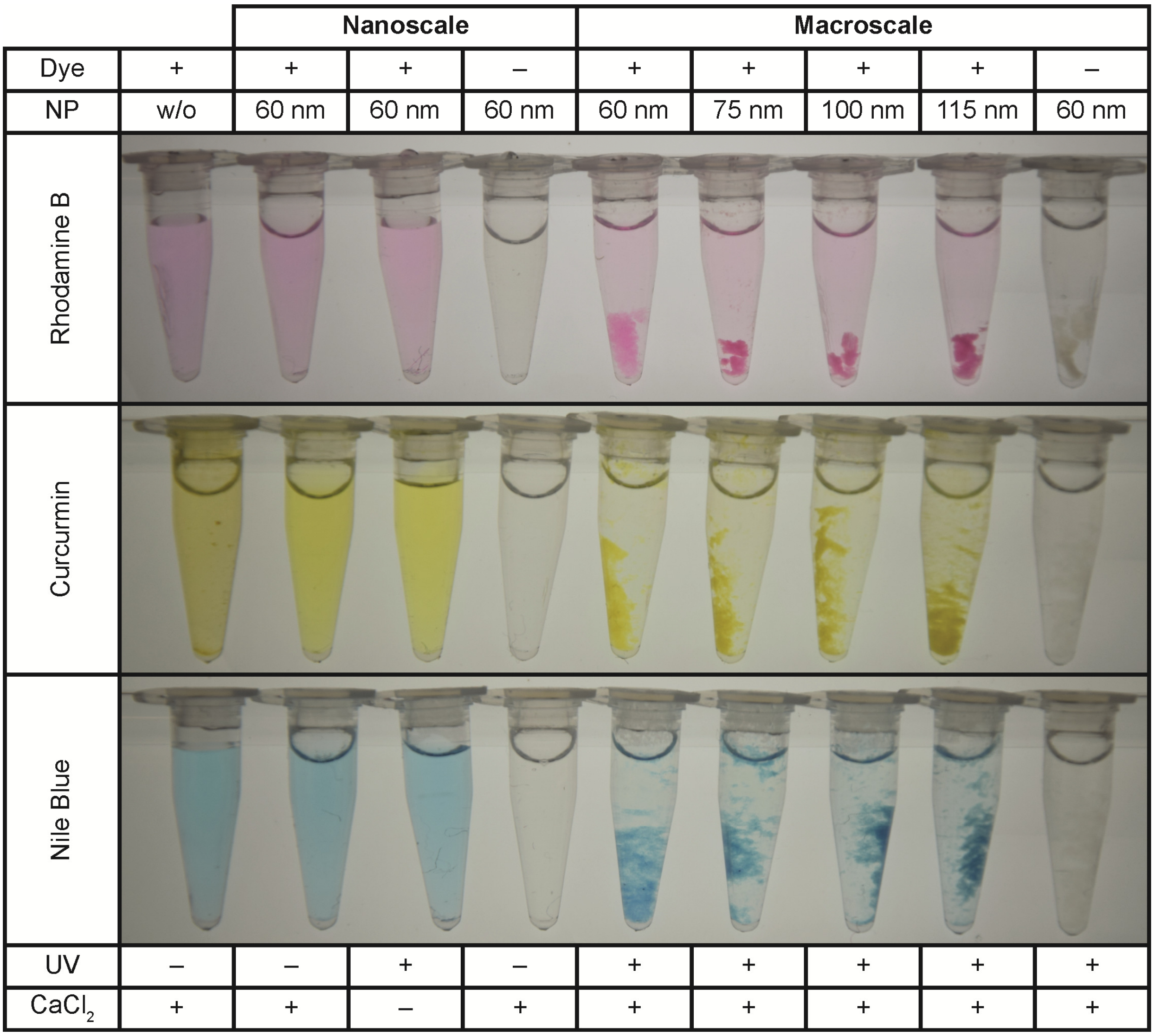

Supplementary Figure 4. The adsorption of dyes onto nanoparticles is evidenced by phase separation. In order to avoid photobleaching of the dyes, the nanoparticles were irradiated prior to incubation with the dyes.

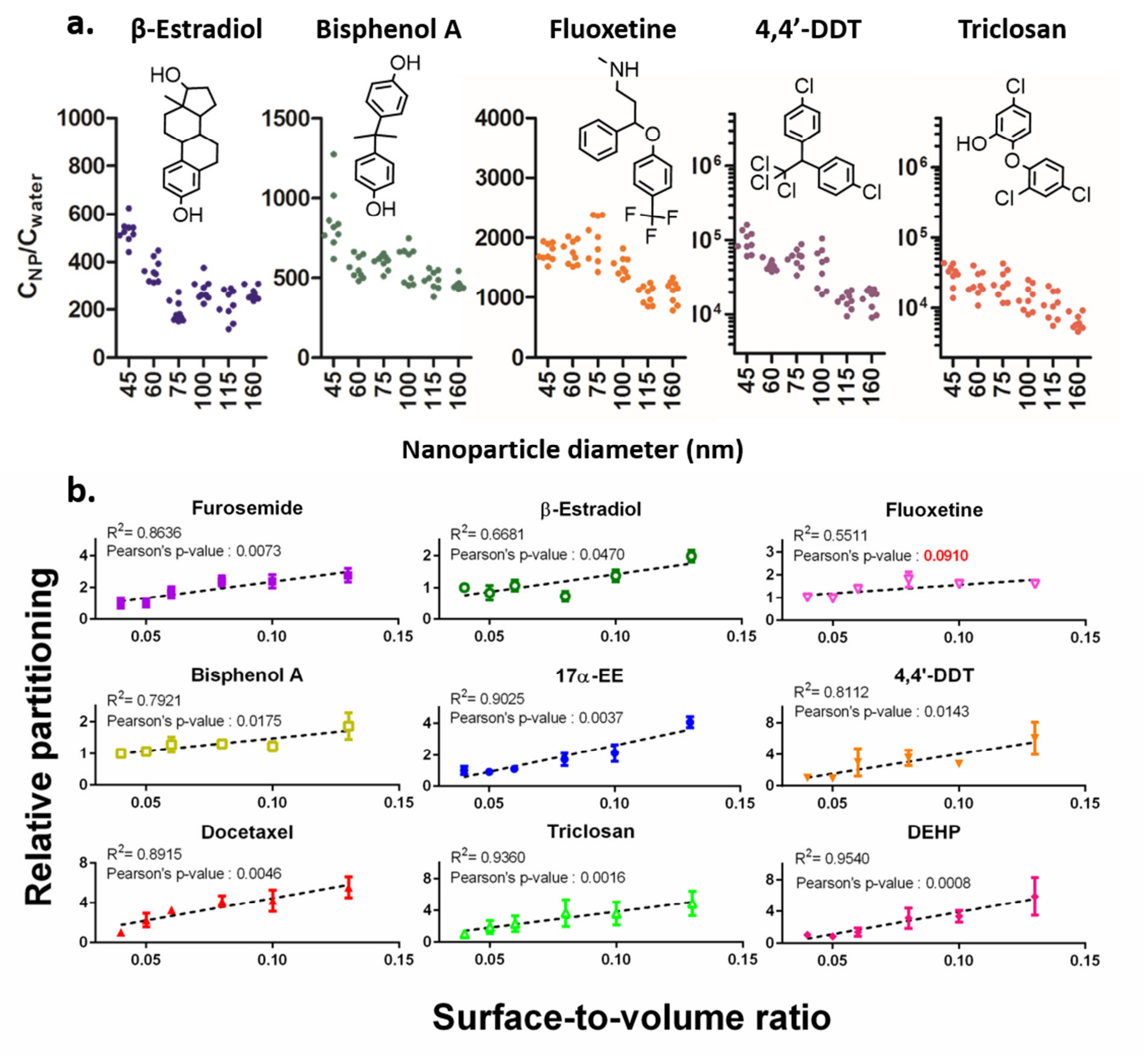

Supplementary Figure 5. A The partitioning into nanoparticles of chemicals with different physicochemical properties appears to increase with decreasing particle size. Values represent each replicate, *n* = 9–12. **B.** The increase in partitioning appears to correlate with an increase in surface-to-volume ratio. To facilitate comparison, partition coefficients for each chemicals were normalized to those obtained with 160-nm nanoparticles. Values represent mean ± standard deviation, *n* = 9–12.

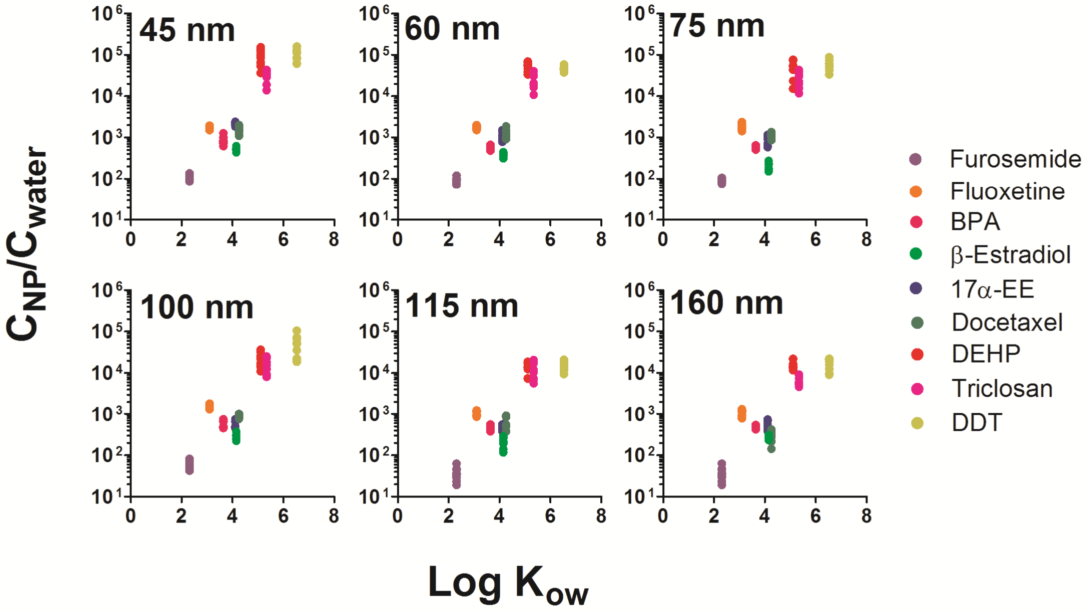

Supplementary Figure 6. For all tested particle sizes, the adsorption onto nanoparticles appears to correlate with the hydrophobicity of the small molecules, suggesting that hydrophobic interactions are at least partly responsible for the interactions between the chemicals and the nanoparticles.

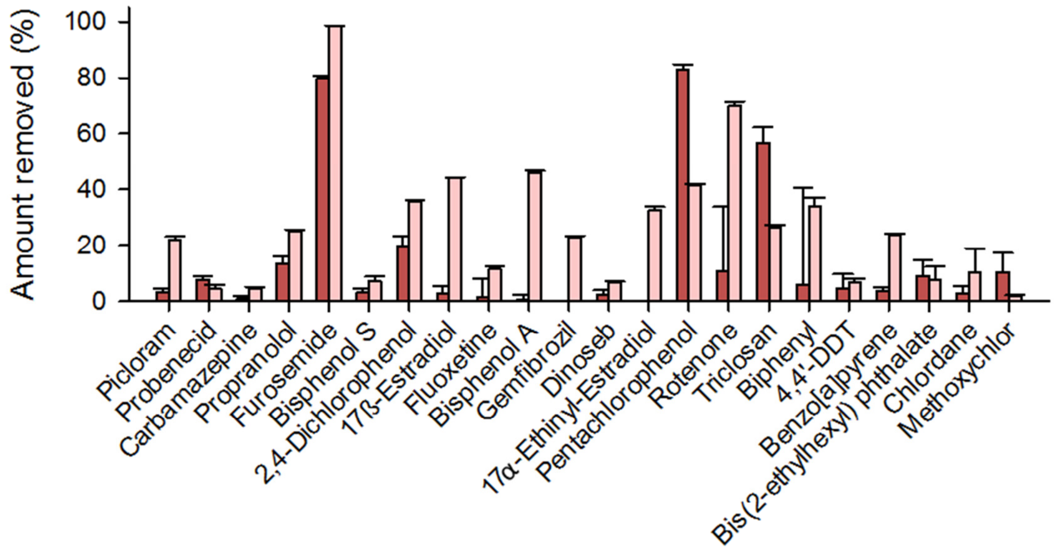

Supplementary Figure 7. In the absence of nanoparticles (dark red), the photo-degradation of most chemicals is reduced, suggesting that the photocleavable linker plays an active role in the reaction. Different degradation mechanisms might be involved since, for some chemicals (*i.e.*, pentachlorophenol, triclosan and methoxychlor), photo-degradation appears to be reduced in the presence of nanoparticles (light pink).

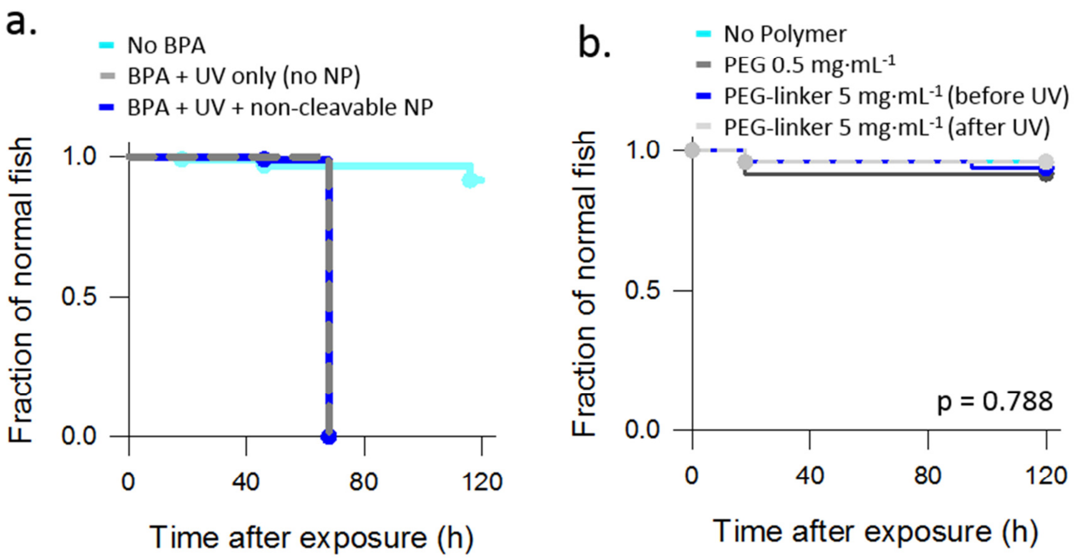

Supplementary Figure 8. A. To show that the reduced teratogenicity observed after treatment with photo-responsive nanoparticles was due to extraction and photo-degradation of BPA, control experiments were carried out in which an equivalent amount of BPA was irradiated with UV light (in gray), or irradiated with UV light in the presence of non-photo-responsive nanoparticles (in dark blue). In both groups, all fish died during the 2nd day post exposure to BPA, while fish not exposed to BPA survived (> 90% survival) ($n = 96$, $p < 0.001$). **B.** Functionalization of PEG with the hydroxyethyl photolinker did not enhance the teratogenicity of the polymer either before or after UV irradiation (> 90% survival). Here, the concentrations of the functionalized polymers were 10 times higher than those of unmodified PEG (log rank, $p = 0.788$, $n = 48$).

a. 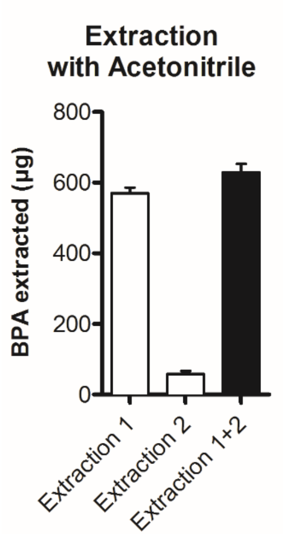

b. 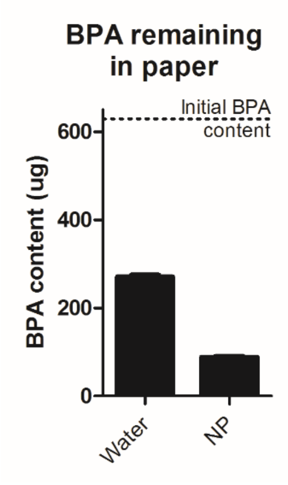

Supplementary Figure 9. A. Amount of BPA extracted from thermal printing paper using acetonitrile. The total BPA content was calculated by adding the values of two consecutive extraction steps. **B**. The amount of BPA remaining in the thermal printing paper is inversely proportional to the amount extracted with water or nanoparticles.

SUPPLEMENTARY TABLES

Supplementary Table 1: Molecular weight and polydispersity of photocleavable diblock copolymers

Copolymer	M_n (NMR)[a]	M_n (GPC)[b]	M_w (GPC)[b]	PDI[c]
PEG5k-*b*-PLA5k	9992	16,679	18,804	1.13
PEG5k-*b*-PLA10k	14,648	17,888	20,978	1.17
PEG5k-*b*-PLA20k	25,184	21,658	25,826	1.19
PEG5k-*b*-PLA35k	39,380	30,264	42,196	1.39

[a] Relative molecular mass determined by ^{1}H-NMR ($CDCl_3$, 300 MHz)
[b] Relative molecular mass determined by GPC against polystyrene standards
[c] Polydispersity index (M_w / M_n)

Supplementary Table 2: Copolymer concentrations used for the different formulations (in mg·mL^{-1}, total polymer concentration 10 mg·mL^{-1}) and representative sizes of the prepared nanoparticles

		PEG$_{5k}$-**linker**-PLA$_{35k}$	PEG$_{5k}$-**linker**-PLA$_{20k}$	PEG$_{5k}$-**linker**-PLA$_{10k}$	PEG$_{5k}$-**linker**-PLA$_{5k}$	PEG$_{5k}$-PLA$_{20k}$	PLGA$_{30k}$	PLGA$_{95k}$	Radioactive PLGA$_{20k}$	Size (mean ± SD)	PDI
**Fig. 1C**	20%		1			4	5			89 ± 0.60	0.114 ± 0.021
	40%		2			3	5			92 ± 1.42	0.169 ± 0.010
	60%		3			2	5			93 ± 0.23	0.132 ± 0.019
	80%		4			1	5			92 ± 1.15	0.149 ± 0.011
	100%		5				5			100 ± 0.57	0.106 ± 0.006
**Suppl. Fig. 1**	25%		2			6	2			59 ± 0.58	0.132 ± 0.019
	50%		4			4	2			62 ± 0.48	0.14 ± 0.009
	75%		6			2	2			64 ± 0.06	0.153 ± 0.006
	100%		8				2			69 ± 0.98	0.156 ± 0.009
**Fig. 1D**	60-nm		4	5.9					0.1	59 ± 0.10	0.129 ± 0.002
	75-nm		7.4				2.5		0.1	83 ± 0.42	0.142 ± 0.010
	100-nm		4.95				4.95		0.1	103 ± 0.68	0.085 ± 0.009
	120-nm		2.5				7.4		0.1	120 ± 1.85	0.093 ± 0.033
**Fig. 2**	45-nm		10							45 ± 0.20	0.147 ± 0.015
	60-nm	2	5.5				2.5			60 ± 1.05	0.149 ± 0.008
	75-nm		7.5				2.5			73 ± 0.40	0.153 ± 0.016
	100-nm		5				5			109 ± 6.51	0.204 ± 0.018
	115-nm		2.5				7.5			117 ± 1.14	0.095 ± 0.010
	160-nm			4				6		164 ± 1.05	0.095 ± 0.005
**Fig. 3**	Panel A	2	5.5				2.5			58 ± 0.62	0.151 ± 0.012
	Panel B	2	5.5				2.5			61 ± 0.52	0.227 ± 0.004
**Fig. 4**	Water	2	5.5				2.5			54 ± 0.46	0.213 ± 0.004
	Thermal paper	2	5.5				2.5			62 ± 0.32	0.152 ± 0.003
	Soil				9			1		89 ± 0.36	0.213 ± 0.005

Supplementary Table 3: HPLC methods for all compounds

Molecule	Injection volume (µL)	Flow (mL·min^{-1})	Acetonitrile (%)	Water 0.1% TFA (%)	Ret. Time (min)	Detector	λ (nm)
**Bisphenol A**	10–20	1	40	60	6.7	FLD	225 / 310
**17α-Ethinyl estradiol**	10–20	1	40	60	9.5	FLD	225 / 310
**17β-Estradiol**	10–20	1	40	60	7.5	FLD	225 / 310
**Bisphenol S**	40	1	20	80	10.1	MWD	254
**Carbamazepine**	40	1	30	70	8.2	MWD	285
**Chlordane**	40	1	70	30	10.8	MWD	210
**DEHP**	20–40	1	85	15	13.0	MWD	210
**4,4'-DDT**	20–40	1	85	15	5.3	MWD	210
**Benzo[a]pyrene**	40	1	85	15	5.8	FLD	230 / 460
**2,4-Dichlorophenol**	40	1	43	57	6.6	MWD	227
**Docetaxel**	20–40	1	43	57	12.5	MWD	227
**Fluoxetine**	20–40	1	40	60	7.2	FLD	230 / 290
**Furosemide**	20–40	1	35	65	6.0	MWD	230
**Gemfibrozil**	40	1	70	30	4.7	FLD	242 / 300
**Picloram**	40	1	25	75	5.6	MWD	230
**Probenecid**	40	1	45	55	7.4	MWD	250
**Propranolol**	10	1	40	60	4.0	FLD	230 / 340
**Rotenone**	40	1	45	55	14.7	MWD	210
**Triclosan**	20–40	1	70	30	5.3	MWD	210
**Methoxychlor**	40	1	70	30	6.1	MWD	210
**Pentachlorophenol**	40	1	70	30	5.0	MWD	210
**Dinoseb**	40	1	70	30	4.8	MWD	280
**Biphenyl**	40	1	70	30	5.0	MWD	210

SUPPLEMENTARY METHODS

Photocleavage of PEG-*b*-PLA copolymers

To investigate the photocleavage of PEG-*b*-PLA copolymers, 20 mg of PEG5k-*b*-PLA10k were dissolved in 2 mL of acetonitrile. The solution was split into two batches; in each case, 1 mL of this solution was dropped into 10 mL of water and stirred over night under light protection to evaporate the acetonitrile. All batches were combined and concentrated to 2 mL by using Amicon® Ultra-15 Centrifugal Filter Units, molecular weight cut-off 100 kDa (EMD Millipore, Billerica, MA). Aliquots of 250 µL were irradiated with UV light (320–395 nm, DYMAX® BlueWave® 200 UV Curing Spot Lamp, Dymax Corporation, Torrington, CT) for 0 s, 5 s, 10 s, 30 s, 60 s, and 300 s; the light intensity was adjusted to 10 $mW{\cdot}cm^{-2}$. The irradiated samples were freeze-dried and dissolved in 500 µL of chloroform. The samples were analyzed by gel permeation chromatography (GPC) on a Shimadzu 10AVP HPLC system equipped with a Shimadzu RID-10A refractive index detector (Shimadzu Deutschland GmbH, Duisburg, Germany) at 40 °C. A Phenogel™ 5 µm 500 Å column was used in combination with a Phenogel™ 5 µm guard column (Phenomenex®, Aschaffenburg, Germany). One hundred µL of each sample were injected; chloroform was used as mobile phase (flow rate 1 $mL{\cdot}min^{-1}$).

Precipitation of nanoparticles in the presence of NaCl or acidic pH

To evaluate the effect of NaCl or different pH values on the precipitation of nanoparticles, 10 µL of [^{14}C]-labeled nanoparticles were added to 80 µL of water, 10 mM acetic acid buffer (pH 4) or 10 mM trifluoroacetic acid buffer (pH 1). The samples were incubated for 10 min, and then supplemented with 10 µL of water, 50 mM $CaCl_2$ or 1.5 M NaCl. Afterwards, the samples were irradiated with UV light for 1 min (320–395 nm, 10 $mW{\cdot}cm^{-2}$, DYMAX® BlueWave® 200 UV Curing Spot Lamp, Dymax Corporation, Torrington, CT). The irradiated samples were then centrifuged for 5 min at 100 *rcf*; then, 20 µL of the supernatant were analyzed by scintillation counting on a Tri-Carb 2810 TR Liquid Scintillation Analyzer (Perkin Elmer, Waltham, MA). The amount of particles remaining in suspension was measured by comparing the measured radioactivity to that in untreated samples. Each experiment was conducted in triplicate.

High performance liquid chromatography (HPLC) quantification

The concentration of chemicals in solution was quantified using reverse-phase HPLC on a 1260 Infinity system (Agilent, Santa Clara, CA) equipped with a quaternary pump (1260 Quat Pump VL GB11C), an autosampler (1260 ALS G1329B), a thermostatted column compartment (1260 TCC G1316A), a multiple wavelength detector (1260 MWD VL G13265D) and a fluorescence detector (1260 FLD G1321B). A C-18 Zorbax 300SB-C18 column, 5 µM, 4.6 x 250 mm was used. The chemicals were separated by isocratic elution using different mixtures of HPLC-grade acetonitrile (Sigma-Aldrich, St-Louis, MO) and 0.1% TFA in ultrapure water (Millipore, Billerica, MA). The mobile phase compositions as well as the detection wavelengths are presented in Supplementary Table 3.
